# Community-based human–elephant conflict mitigation: The value of an evidence-based approach in promoting the uptake of effective methods

**DOI:** 10.1371/journal.pone.0173742

**Published:** 2017-05-16

**Authors:** Donny Gunaryadi, Sugiyo, Simon Hedges

**Affiliations:** 1 Wildlife Conservation Society – Indonesia Program, Bogor, Indonesia; 2 Wildlife Conservation Society – Global Conservation Programs, Bronx, New York, United States of America; U.S. Geological Survey, UNITED STATES

## Abstract

Human–elephant conflict (HEC) is a serious threat to elephants and can cause major economic losses. It is widely accepted that reduction of HEC will often require community-based methods for repelling elephants but there are few tests of such methods. We tested community-based crop-guarding methods with and without novel chili-based elephant deterrents and describe changes in farmers’ willingness to adopt these methods following our demonstration of their relative effectiveness. In three separate field-trials that took place over almost two years (October 2005 –May 2007) in two villages adjacent to Way Kambas National Park (WKNP) in Indonesia, we found that community-based crop-guarding was effective at keeping Asian elephants (*Elephas maximus*) out of crop fields in 91.2% (52 out of 57), 87.6% (156 out of 178), and 80.0% (16 out of 20) of attempted raids. Once the method had been shown to be effective at demonstration sites, farmers in 16 villages around WKNP voluntarily adopted it during the July 2008 to March 2009 period and were able to repel elephants in 73.9% (150 out of 203) of attempted raids, with seven villages repelling 100% of attempted raids. These 16 villages had all experienced high levels of HEC in the preceding years; e.g. they accounted for >97% of the 742 HEC incidents recorded for the entire park in 2006. Our work shows, therefore, that a simple evidence-based approach can facilitate significant reductions in HEC at the protected area scale.

## Introduction

Species conservation will be more effective if it is based on good science and reliable evidence but too often this is not the case [1, 2]. While there is a growing appreciation of the dangers of making interventions without evidence of their effectiveness, this appreciation is growing too slowly and is failing to have sufficient impact on conservation practice, even for high profile species such as Asian elephants (*Elephas maximus*) and African elephants (*Loxodonta africana*) [3–5]. Moreover, as Hall and Fleishman [6] argue, failure to evaluate under field conditions how a method performs or the cost of its implementation can prevent its acceptance by end users. There is, therefore, a pressing need for both evidence-based approaches and for such approaches to be linked to practical demonstrations, which are defined by Hall and Fleishman as the “translation of scientific [research] into metrics of performance and cost of implementation under real-world conditions”.

Human–elephant conflict (HEC) has been identified as one of the most serious threats to elephants [7], not least because where elephants persist they are often forced into close contact with people and contemporary social conditions often lower people’s tolerance of elephants [8]. To avoid retaliatory killing of elephants, conservationists need to increase people’s tolerance of elephants, and this will require reducing the impact of crop depredations. While it is widely-believed that sustainable reduction of HEC will require, in many cases, community-based methods for repelling elephants (see for example [9, 10]) there are few tests of such methods’ effectiveness.

Elephants were once widespread throughout Sumatra’s Lampung Province but are now restricted to three areas: Bukit Barisan Selatan National Park, Way Kambas National Park, and the Gunung Rindingan–Way Waya protected forest complex [11]. Human–elephant conflict, particularly crop depredation, is a major problem in Lampung, as it is in much of the rest of Sumatra, and results in retaliatory killing of elephants and pressure on the authorities to remove elephants from the wild [11–13]. Effective mitigation of human–elephant conflict is therefore an essential part of efforts to conserve Sumatra’s elephants, which are listed as Critically Endangered in the *IUCN Red List* [14].

In the study reported here, we tested both conventional crop-guarding methods and chili-based elephant deterrents (chili grease smeared on rope fences): these methods were chosen because (1) they are widely-used in Asia and Africa (with varying degrees of success) and are included in numerous manuals on how to reduce HEC (see for example [15, 16]) and (2) the conventional crop-guarding methods were traditionally used, albeit with limited success, by farmers around WKNP. In an earlier publication, we showed that it was possible to keep elephants out of crop areas using conventional unsophisticated tools and guarding techniques, and that chili-based methods did not add any further deterrent value to that provided by the conventional methods but did add expense and created additional work [17]. However, the beneficial effects were demonstrated in just two small areas on the border of the national park. Here, we describe additional tests of the chili-based and conventional methods and show: (1) that the combination of simple early warning systems to detect elephants before they have entered crop fields together with a communal approach to guarding using simple tools is indeed an effective method for reducing crop raiding by elephants; (2) that even farmers not involved in the original tests will voluntarily adopt such methods if the methods’ effectiveness is demonstrated through the provision of readily understandable evidence; (3) that, most importantly, a simple evidence-based approach of this type can achieve significant reductions in crop raiding rates at the protected area scale rather than at just the village scale.

## Methods

### Study area

**Fig 1:**
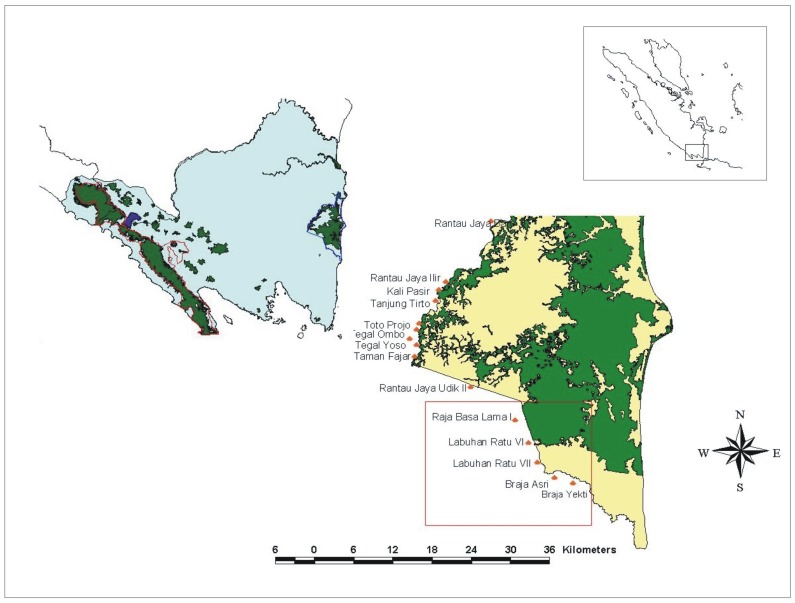
Project area in and around Way Kambas National Park (WKNP), Sumatra, Indonesia. The top right panel depicts the island of Sumatra in relation to Peninsular Malaysia and Java, with the study area in a box. The left panel depicts the boxed area in greater detail with remaining forest cover shown in green. The bottom right panel shows WKNP and the 16 villages mentioned in the text. Figure used by permission of the Wildlife Conservation Society (WCS), original copyright owner [original copyright year 2007], under a CC BY license.

The study was conducted around Way Kambas National Park (WKNP) in Lampung Province, Sumatra, Indonesia (Fig 1). The park covers 1,235 km^2^, is entirely below 50 m asl, and much of it is covered in grassland and scrub. The area adjacent to the park is dominated by settled agriculture, primarily irrigated rice fields and areas cultivated with cassava, maize, and bananas; there is no buffer zone between the park and the agricultural area. The park experiences a mean annual rainfall of about 2,000 mm and a pronounced dry season of 2–6 months between March and September [12, 18, 19]. There were an estimated 180 (95% CI = [144, 225]) elephants in WKNP in 2002 [11] with the population estimate derived using standard dung count based methods [20]. No data on whether elephant population size had changed between the 2002 survey and the HEC mitigation work of 2006–2009 described in the present paper are available but we note that in other work we showed that crop raiding occurred along the park’s boundaries in areas adjacent to both high and low elephant density [11]. There were typically some 200–750 HEC incidents per year around the park during 2000–2006, with no obvious trends in HEC rates per year over that period [13, 17, 19]. The number of HEC incidents per site and how those numbers varied with site characteristics was also examined in earlier work [13, 19]. Our work in and around WKNP was conducted under the terms of a Memorandum of Understanding between the Wildlife Conservation Society (WCS) and the Indonesian Government’s Ministry of Forestry, which is the agency responsible for Indonesia’s national parks.

### Crop protection methods tested

The community-based approaches to crop defense that we tested are detailed by Hedges and Gunaryadi [17]. In brief, we evaluated coordinated guarding with and without the addition of chili-based deterrents plus tripwire-triggered sirens. At the ‘conventional’ sites, crop defenses consisted of watchtowers each manned by 2–3 crop guards per night. The watch towers were built close to regular elephant entry/exit points (known as ‘active routes’). The crop guards were equipped with spotlights and some of them had 2-way radios. In addition, crop guards had traditional ‘carbide-canons’ (bamboo tubes in which carbide-generated-acetylene is exploded) which were used to scare any elephants approaching crop fields. When elephants were detected approaching the test sites, the crop guards actively chased the elephants away from the fields and back into WKNP. Barbed wire and rope fences were strung across active routes and these fences had tin-cans-and-stones noisemakers attached in order to alert the guards when elephants approached. Kerosene lamps were also placed by active routes as experience had suggested that these might have a deterrent effect.

At the ‘chili-and-sirens’ sites, the methods and tools were the same as at the conventional sites except (1) there were no tin-can-and-stones noisemakers—these were replaced with trip-wire fences that triggered electronic sirens and (2) chili powder mixed with engine grease (‘chili-grease’) was also applied to rope fences and to cloth spacers attached to these fences, as well as to barbed wire fences running parallel to the cloth fences (Table 1). The chili-grease was made using the hottest chilies available in Lampung Province. It is important to note that Osborn, Parker, and others argue that chili-based deterrents should not be expected to work in isolation but should be deployed as part of a combined approach to crop defenses as in this study [15, 16, 21].

**Table 1:** Crop protection methods used in the ‘conventional’ sites and the ‘chili-and-sirens’ sites in Phases 1 and 2.

Conventional sites	Chili-and-sirens sites
Guards in watch towers with spotlights and 2-way radios	Guards in watch towers with spotlights and 2-way radios
Noise-makers	Noise-makers
Kerosene lamps	Kerosene lamps
Tin-cans-and-stones alarm fences	Sirens+tripwire alarm fences
	Chili-grease fences

In Phase 1 (22 October 2005 to 5 April 2006), we selected the Labuhan Ratu VI / Labuhan Ratu VII area, a previously identified HEC ‘hotspot’ on the border of WKNP, for our tests. Community-based crop-guarding with conventional tools was deployed at the ‘conventional site’, with community-based crop-guarding plus chili-based deterrents and trip-wire triggered sirens at a contiguous site (the ‘chili-and-sirens site’). The two sites covered a total area of 275.8 ha and contained three crop types: cassava, rice, and a very small amount of maize (Table 2). All three crop types were known to be consumed by elephants around WKNP [12, 19]. The chili-and-sirens site had fewer towers than the conventional site because (i) a road and houses formed its northern border and (ii) there were fewer elephant active routes into the chili-and-sirens site.

**Table 2:** Characteristics of the Labuhan Ratu sites in Phases 1 and 2 and the Braja Asri sites in Phase 2.

Site name and period	No. of towers	Boundary length (km)	Cassava (ha)	Rice (ha)	Maize (ha)	Water-melon (ha)	Tall grass (ha)	Others (ha)	Total test area (ha)
**Phase 1 (22 October 2005 to 5 April 2006)**									
Labuhan Ratu chili-and-sirens site	4	2	90.6	3.0	0	0	0	0	93.6
Labuhan Ratu conventional site	8	2	173.5	7.5	1.1	0	0	0	182.1
**Labuhan Ratu totals (Phase 1)**	**12**	**4**	**264.2**	**10.5**	**1.1**	**0**	**0**	**0**	**275.8**
**Phase 2 (17 January to 12 May 2007)**									
Labuhan Ratu new conventional site*	10	4	237.1	20.6	0	12	0.75	5.35	275.8
**Labuhan Ratu totals (Phase 2)**	10	4	237.1	20.6	0	12	0.75	5.35	275.8
Braja Asri chili-and-sirens site	5	1.7	0	55.0	0	0	0	0	55.0
Braja Asri conventional site	5	1.9	0	64.9	0	0	0	0	64.9
**Braja Asri totals (Phase 2)**	**10**	**3.6**	**0**	**119.9**	**0**	**0**	**0**	**0**	**119.9**

* = Former conventional site plus former chili-and-sirens site.

In Phase 2 (17th January until 12th May 2007), we again compared a ‘conventional site’ and a ‘chili-and-sirens site’ but in a different area, Braja Asri. We selected Braja Asri because it was dominated by rice (unlike the Labuhan Ratu area, which is dominated by cassava) and it had been identified as a HEC ‘hotspot’. In addition, Braja Asri is a swampy area, and we were interested in assessing the feasibility of protecting crops in such areas. In Phase 2, we again conducted a trial in the same Labuhan Ratu sites used in Phase 1. During Phase 2, however, the number of towers was reduced from 12 to 10 because two of the original towers were deemed to be too far from the WKNP border (Table 2). Sites were allocated at random to either the chili-and-sirens methods or the conventional methods in Phase 1 and 2, and the trial period in both phases coincided with the main period of crop-growing in Labuhan Ratu and Braja Asri.

A key consideration in the design of the study was to ensure that none of the methods used would result in injuries to people or elephants. To that end, none of the methods used involved any direct contact with elephants (e.g. no projectiles were aimed at elephants). All methods used in the study are widely used in Asia and Africa and are included in standard manuals on the reduction of HEC (see for example [15, 16]). Furthermore, any limited stress that could be caused to the elephants on the occasions when they were actively driven away from crops was judged to be outweighed very significantly by the reduction in threats to the elephant population (e.g. reduced numbers of retaliatory killings or capture and removal from the wild) that would result from a successful reduction in HEC rates around the park. No people, elephants, or other wildlife were injured or harmed during the study.

### Villager participation

During Phase 1, we hired villagers to act as crop guards, paying them on a full-time basis, because we wanted to be able to effectively compare community-based crop-guarding schemes with and without chili-based deterrents and in the previous two years of work around WKNP we had been unsuccessful in our attempts to initiate community-based crop-guarding on a voluntary basis. We had provided farmers with tools (e.g. sirens, spotlights, and chilies) and helped them build watchtowers but the farmers rarely guarded their crops. In Phase 1, we also initiated and fostered village ‘self-reliance groups’ (*Kelompok Swadaya Masyarakat*, *KSM*) in villages around WKNP to help encourage the adoption of sustainable (voluntary) HEC reduction methods in the future. These self-reliance groups provided opportunities for farmers to discuss HEC and acted as fora for providing informal training in crop guarding and safe elephant driving techniques. The training emphasized the safety of people and elephants and other wildlife. Verbal rather than written consent was obtained from the villagers who participated in the trials; written consent was not obtained because many farmers were illiterate. The consent process was documented and monitored by the self-reliance groups described above.

In Phase 2, no crop guards were paid but the project supplied tools for the guards in the Braja Asri area. In addition, the Labuhan Ratu farmers used the tools supplied in Phase 1 during Phase 2. In Phase 2, all farmers received supplies (carbide and kerosene) as they had during Phase 1. Finally, in Phase 3 (3 July 2008 to 25 March 2009) no crop guards were paid and no tools or supplies were provided (Table 3).

**Table 3:** Support provided by the HEC project to villagers during the three project phases discussed in the text.

Support provided	Labuhan Ratu area, Phase 1 (22 October 2005 to 5 April 2006)	Labuhan Ratu area, Phase 2 (17 January to 12 May 2007)	Braja Asri area, Phase 2 (17 January to 12 May 2007)	Perimeter of WKNP, Phase 3 (3 July 2008 to 25 March 2009)
Paying crop guards	Yes	No	No	No
Providing food for guards	Yes	No	No	No
Providing carbide for noisemakers	Yes	Yes	Yes	No*
Providing kerosene	Yes	Yes	Yes	No*
Providing material for building towers	Yes	No	Yes	No
Charging the batteries	Yes	No	No	No
Fixing the broken watchtowers	Yes	No	No	No

* = In Braja Asri carbide and kerosene were provided if farmers requested it, in all other sites neither carbide nor kerosene were supplied.

### Crop damage assessment

We established Problem Animal Recorder (PAR) teams to collect data on HEC around WKNP throughout the entire study period. The PAR teams visited the villages around WKNP approximately twice a month. In addition, we developed a network of local village ‘informants’ who notified our teams when crop-raiding incidents occurred so as to facilitate rapid evaluation of incidents. Each incident was assessed to see whether it constituted an independent event, which we defined as a single foray occasion, i.e. when an elephant or group of elephants crossed a park boundary, entered farmland, and damaged crops [22]. The date and time of all incidents were noted, as were the crop types damaged, and the location using a Global Positioning System (GPS).

### Assessing the effectiveness of the defenses

We assessed the effectiveness of the crop defenses by recording the total number of elephant crop raiding attempts (“challenges”) along the defended agriculture/elephant habitat interface and the number of attempted raids that were repelled (compare [23]). Monitoring of challenges was conducted every night, from before dusk to after dawn, by the crop guards and our field technicians, with assistance from our PAR teams.

## Results

### Number of elephant challenges to crop defenses and the proportion repelled successfully

During 140 continuous nights of guarding at the Labuhan Ratu sites in Phase 1 there were 34 attempts by elephants to enter crop fields at the chili-and-sirens site, of which 31 (91.2%) were successfully repelled and 57 attempts by elephants to enter crop fields at the conventional site, of which 52 (91.2%) were successfully repelled (for further details see [17]). In Phase 2, during 89 continuous nights of guarding at the Braja Asri sites, there were 62 attempts by elephants to enter crop fields at the chili-and-sirens site, of which 34 (54.8%) were successfully repelled and 20 attempts by elephants to enter crop fields at the conventional site, of which 16 (80.0%) were successfully repelled (Table 4); this difference was significant at *P* = 0.05 (two sample t-test between proportions).

**Table 4:** Comparison of the effectiveness of the two different crop protection systems tested: Community-based crop-guarding with conventional tools (at the ‘conventional’ sites) and community-based crop-guarding with chili-grease fences and trip-wire triggered sirens (the ‘chili-and-sirens’ sites) in Phases 1 and 2.

Site	Number of guarding nights	No. of attempted elephant raids	No. of raids not repelled	No. of raids repelled	Proportion of raids repelled	Source
**Phase 1**						
Labuhan Ratu chili-and-sirens site	140	34	3	31	91.2%	Ref. [17]
Labuhan Ratu conventional site	140	57	5	52	91.2%	Ref. [17]
**Phase 2**						
Labuhan Ratu new conventional site*	115	178	22	156	87.6%	Ref. [17]
Braja Asri chili-and-sirens site	89	62	28	34	54.8%	This study
Braja Asri conventional site	89	20	4	16	80.0%	This study

* = Former chili-and-sirens plus conventional sites combined.

Thus, in Phase 1, the same proportion (91.2%) of attempted raids was repelled at both the conventional and chili-and-sirens sites and there was no apparent difference in the effectiveness of the methods used at these two sites. In Phase 2, by contrast, a larger proportion of attempted raids was repelled at the conventional site than at the chili-and-sirens site (88.0% and 54.8%, respectively).

In Phase 2, farmers in both the former chili-and-sirens and conventional sites at Labuhan Ratu decided to voluntarily adopt the conventional crop defense methods; they did not adopt the chili-and-sirens method. During the 115-night period of voluntary guarding at this site in Phase 2, 87.6% (156 out of 178) of attempted raids by elephants were successfully repelled (Table 4). Furthermore, we observed that the farmers’ confidence had increased compared to Phase 1 and they were prepared to plant more high-risk crops such as rice and watermelons (high-risk because they are favored by elephants); e.g., the area under rice increased by almost 100% (from 10.5 ha to 20.6 ha; Table 2).

In Phase 3, following encouragement by the village self-reliance groups including trips by villagers from around WKNP to the test sites used in Phases 1 and 2, 16 villages voluntarily adopted the methods used in the conventional sites in Phases 1 and 2. No farmers in Phase 3 adopted chili-based methods. The 16 villages had all experienced high levels of HEC in the preceding years; e.g. they accounted for >97% of the 742 HEC incidents recorded for the entire park in 2006. During the 265-night period, there were a total of 203 attempted raids by elephants of which 150 (73.9%) were repelled successfully, with nine of the villages achieving success rates >90% (Table 5).

**Table 5:** Proportion of attempted elephant raids repelled by villagers using voluntary community-based crop protection methods (‘conventional’ methods) for 16 villages in Phase 3.

Village	No. of attempted elephant raids (3 July 2008 to 25 March 2009)	No. of raids not repelled	No. of raids repelled	Proportion of raids repelled successfully	Source
Braja Asri	43	9	34	79.1%	This study
Braja Yekti	7	0	7	100.0%	This study
Labuhan Ratu IX	7	3	4	57.1%	This study
Labuhan Ratu VI	40	17	23	57.5%	This study
Labuhan Ratu VII	19	10	9	47.4%	This study
Muara Jaya	6	4	2	33.3%	This study
Raja Basa Lama I	10	5	5	50.0%	This study
Rantau Jaya Udik II	5	2	3	60.0%	This study
Sidodadi	1	0	1	100.0%	This study
Taman Fajar	11	1	10	90.9%	This study
Tambah Dadi	3	0	3	100.0%	This study
Tanjung Kesuma	3	0	3	100.0%	This study
Tanjung Tirto	2	0	2	100.0%	This study
Tegal Ombo	19	0	19	100.0%	This study
Tegal Yoso	24	2	22	91.7%	This study
Totoprojo	3	0	3	100.0%	This study
**Grand total**	**203**	**53**	**150**	**73.9%**	

## Discussion

### Our results

In three tests during Phases 1 and 2, we found that community-based crop-guarding methods using conventional tools without chili-based deterrents were effective at keeping elephants out of crop fields in 91.2% (52 out of 57), 87.6% (156 out of 178), and 80.0% (16 out of 20) of attempted raids [17]. Most importantly, as shown by the additional work reported in this paper, once the conventional methods had been shown to be effective in Phases 1 and 2, farmers in 16 HEC “hotspot” villages around WKNP voluntarily adopted them in Phase 3 and were able to repel elephants successfully in 73.9% (150 out of 203) of attempted raids, with seven villages repelling 100% of attempted raids.

For the comparison between the crop protection methods in Phase 1 both the conventional site and the chili-and-sirens site were predominantly under cassava (95.28% and 96.79% of the crops grown by area, respectively; Table 2) and for the comparison between the methods in Phase 2 both the conventional site and the chili-and-sirens site were both solely planted with rice (Table 2): so, in effect both sites were identical in terms of the crops grown. There is no reason, therefore, to suspect that the amount and type of crops grown influenced the results in Phase 1 or Phase 2.

It is important to note that the results of our tests of the crop protection methods in Phases 1 and 2 could not tell us whether the crop protection methods were likely to be effective for maize or other crop types such as bananas commonly grown around WKNP given that the test sites in Phases 1 and 2 were effectively just under rice and cassava. However, from earlier work conducted by the PARs around WKNP, we knew that conventional crop protection methods can be effective at protecting maize and many others crop types [19]. Furthermore, given that significant if un-mapped (because of the size of the area under crops around the perimeter of WKNP) quantities of maize and bananas were grown in the crop fields of the 16 villages successfully deploying the conventional crop-guarding methods in Phase 3, the methods do seem in practice to protect maize and banana crops as well as rice and cassava.

We had previously shown that the addition of chili-grease fences to the crop defense systems deployed did not add any apparent deterrent effect in Phase 1 (as explained in [17]); what was unexpected in the Phase 2 work reported in this paper was that the addition of chili-grease fences would be associated with a reduction in the effectiveness of the crop defenses when compared to the contiguous ‘conventional’ test site. One possible explanation for the apparent reduction in the effectiveness of the crop defense system in the Phase 2 chili-and-sirens test site could be that the farmers were less vigilant because they thought the chilies would keep elephants away but the PAR teams did not assess the farmers’ vigilance and so that possible explanation, while plausible, must be treated as speculation. Note that early-warning systems were deployed in all conventional and chili-and-siren sites, so the smaller proportion of elephant raids that were repelled at the chili-and sirens site in Phase 2 is unlikely to be a result of the use of siren-based alarm systems in that site.

### Villager participation

Prior to the work described here and an earlier paper [17], farmers around WKNP were unenthusiastic about participating in our earlier voluntary trials of crop defenses and so we had been unsuccessful in our attempts to encourage sufficiently intense guarding systems (for similar observations see [24]). This lack of enthusiasm was in part due to a—declared—lack of faith in the efficacy of crop guarding systems because of the poor success rates the farmers had experienced in the past. These poor success rates were due primarily to low and erratic participation rates by farmers; e.g. on most nights, too few farmers had guarded their crops and so detection and scaring-away of elephants were both compromised leading to the area’s farmers concluding erroneously that crop-guarding methods were always ineffective (authors’ pers obs). We therefore hired villagers (not necessarily farmers) to act as guards in Phase 1 so that we could ensure the watchtowers were manned and thus effectively compare community-based crop-guarding schemes with and without chili-based deterrents.

However, since we were primarily interested in promoting a sustainable approach to HEC mitigation around WKNP, we only paid crop guards in Phase 1. Moreover, we made clear our intention to pay for crop guards for one season only from the start of Phase 1. Once the Phase 1 tests were complete, we concentrated on promoting self-reliance and voluntary guarding through the creation of the self-reliance groups, by explicitly treating Phase 2 as a demonstration, and by means of a series of village meetings around WKNP in 2006–2008 that included organized visits to the Phase 1 and 2 trial sites.

### Prevention of human–elephant conflict *versus* repelling attempted crop depredations by elephants

The best approach to HEC is to prevent it in the first place through, for example, land use planning that does not allow the growing of crops attractive to elephants adjacent to elephant habitat. However, the unfortunate reality in most elephant range areas is that the opportunities for such ‘elephant-sensitive’ land use planning were lost long ago. Consequently, there are many areas like WKNP and its environs where crops are grown right up to the edge of elephant habitat and the crops are often poorly guarded or not guarded at all because the farmers have had too many years of experiencing failed defense systems, as at WKNP prior to the trials described in this paper. In certain circumstances, electric fencing along the agriculture/elephant habitat interface can be appropriate and electric fences around privately-owned cultivated lands have reportedly achieved significant successes in India [25], for example, while a success rate of 80% has been reported for electric fences around oil palm and rubber plantations in Malaysia [7]. Nevertheless, the use of fencing for wildlife management including HEC mitigation has attracted considerable controversy in recent years [26–30], in part because of the inherent risks of population fragmentation. Furthermore, because the damage done by crop raiding elephants may not be that significant in direct economic terms (e.g. for the 20 villages around WKNP that had HEC problems between June 2000 and May 2002, the direct financial loss due to crop raiding was only about US$12,000 for that period [11]) notwithstanding the opportunity costs associate with HEC, which can be high, it is often hard to justify the expense of building electric fences or other physical barriers. For example, a 6-strand electric fence would cost approximately US$9000/km to construct and would also require regular, often costly, maintenance. There is, therefore, a significant need across elephant range countries for effective, low-cost (and thus potentially sustainable) methods for keeping elephants out of agricultural land that can be easily implemented by the farmers themselves (thus facilitating self-reliance)–methods such as those described in this paper.

### Conclusions and recommendations

This study goes beyond our earlier work [17] in that it further calls into question the use of chili products as elephant deterrents, since chili-grease fences were associated with a reduction in the effectiveness of the crop defense systems in the additional tests reported in this paper (chili-grease fences had contributed no apparent deterrent effect in our earlier tests of crop defenses systems [17]). Clearly, further tests of chili-based methods should be conducted. Encouragingly, however, our trials, like those of Osborn and Parker [31], Sitati and Walpole [23], and Davies *et al*. [32], show that a combination of simple early warning systems to detect elephants before they have entered crop fields together with a communal approach to guarding using simple tools is an effective method for reducing elephant crop raiding rates. Most importantly, our work shows that farmers will voluntarily adopt such methods if the methods’ effectiveness is demonstrated through the provision of readily understandable evidence (proportions of attempted elephant raids repelled in this case) and that a simple evidence-based approach of this type can achieve significant reductions in crop raiding rates at the protected area scale rather than at just the village scale. Finally, our work lends supports to the conclusion of Hall and Fleishman [6] that the participation of end users in the development and execution of demonstration projects helps ensure that performance measures are credible and increases the probability that successful innovations will be adopted.

A large proportion of conservation interventions are based on experience, expert opinion, or even anecdote, not evidence [2, 33]. This unfortunate situation is in part due to the difficulties—and potential ethical problems—inherent in conducting experiments in field situations on issues which threaten human lives and livelihoods [9, 32]. However, our work has shown that the effectiveness of interventions aimed at reducing crop depredations by elephants can be assessed from simple field trials, and the results of such trials can be used to encourage the uptake of effective methods with increased support from the wider community.

More generally, it is essential to ensure that interventions aimed at reducing human–wildlife conflict (HWC) are promoted only if there is evidence of their effectiveness because: (1) it helps improve the take-up of effective methods for reducing HWC; (2) it will help prevent people having to continually ‘reinvent the wheel’ (or worse, continually testing ‘square wheels’ [i.e. ineffective methods]); and (3) it is unethical to promote methods that have not been properly tested, especially if use of such methods involves significant investment of time and/or money given that HWC disproportionately affects the poorest segments of society.
